# *Staphylococcus aureus* Myocarditis with Associated Left Ventricular Apical Thrombus

**DOI:** 10.1155/2018/7017286

**Published:** 2018-05-23

**Authors:** Michael McGee, Emily Shiel, Stephen Brienesse, Stuart Murch, Robert Pickles, James Leitch

**Affiliations:** ^1^Cardiovascular Department, John Hunter Hospital, Lookout Road, New Lambton Heights, NSW 2305, Australia; ^2^Infectious Diseases Department, John Hunter Hospital, Lookout Road, New Lambton Heights, NSW 2305, Australia; ^3^Department of Medicine, University of Newcastle, Newcastle, NSW, Australia

## Abstract

*Staphylococcus aureus* myocarditis is a rare diagnosis with a high mortality rate, usually seen in people who are immunocompromised. Here, we report a case of a 44-year-old man on methotrexate for rheumatoid arthritis who presented in septic shock and was diagnosed with *staphylococcus aureus* myocarditis. The myocarditis was associated with a left ventricular apical thrombus, with normal systolic function. The myocarditis and associated thrombus were characterised on transthoracic echocardiogram and subsequently on cardiac magnetic resonance imaging. Cardiac magnetic resonance (CMR) imaging showed oedema in the endomyocardium, consistent with acute myocarditis, associated with an apical mural thrombus. Repeat CMR 3 weeks following discharge from hospital showed marked improvement in endomyocardial oedema and complete resolution of the apical mural thrombus. He was treated with a 12-week course of antibiotics and anticoagulated with apixaban. The patient was successfully managed with intravenous antibiotics and anticoagulation with complete recovery.

## 1. Introduction

*Staphylococcus aureus* is widely reported to be the most common bacterial cause of myocarditis and usually occurs in the setting of bacteraemia and sepsis. Rarely, it occurs without associated infective endocarditis [1, 2]. Furthermore, difficulty in determining the diagnosis, prevalence, and aetiology of myocarditis is complicated by the infrequent use of endomyocardial biopsy (EMB), the diagnostic gold standard [2, 3].

Noninvasive imaging such as cardiac magnetic resonance (CMR) imaging has been shown to be reliable in the diagnosis and monitoring of disease progression in acute myocarditis [4, 5]. Despite the advances of noninvasive imaging, the sensitivity and specificity of CMR for acute myocarditis are reported at 81% and 71% and 63% and 40% in chronic myocarditis [6]. Ideally, CMR and EMB are both obtainable and are complimentary, overcoming limitations of either technique alone [4].

Here, we present a case of *Staphylococcus aureus* myocarditis with associated left ventricular apical thrombus.

## 2. Case History

A 44-year-old male was admitted to the intensive care unit (ICU) critically unwell with septic shock. On the day prior to admission, he had complained of lethargy, diffuse body aches, epigastric pain, nausea, and vomiting and had experienced rigors. He had a medical background significant for rheumatoid arthritis, treated with methotrexate 10 mg weekly for the last five years and had recently travelled to Fiji.

On arrival of paramedics, the patient was nonresponsive, febrile, and tachycardic, with an unrecordable blood pressure and mottled appearance of the skin. He was drowsy and confused on arrival to the emergency department and had prolonged capillary refill of five seconds, a lactate of 6.8. There was no clear focus of infection on examination, and cardiovascular examination was normal at this time. He was found to have an acute kidney injury, hepatic impairment, and coagulopathy, see Table 1. Blood cultures returned positive in 11 hours for methicillin-sensitive *Staphylococcus aureus*, and antibiotics were changed to intravenous (IV) flucloxacillin. He was culture positive for 48 hours.

**Figure 1:**
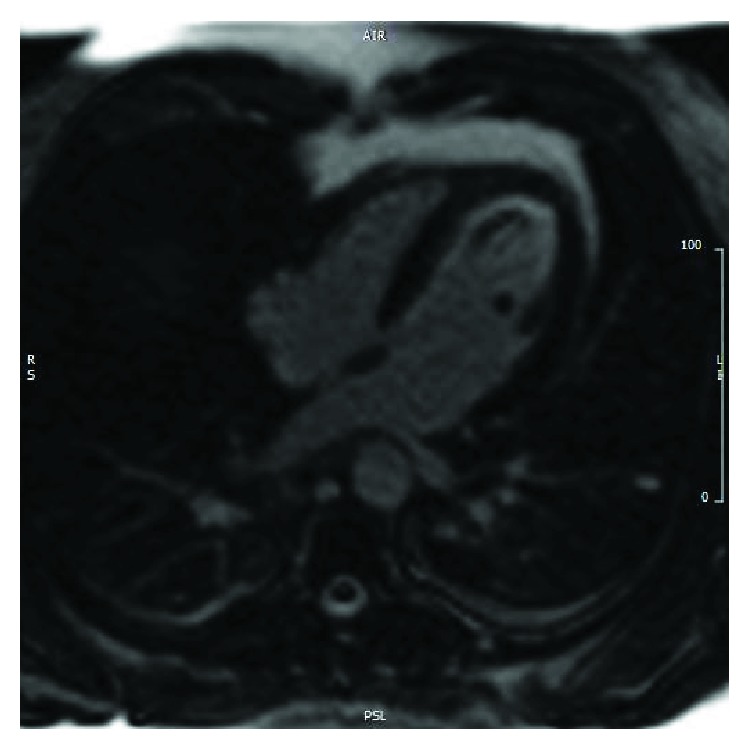
Cardiac magnetic resonance imaging 4-chamber-view post gadolinium injection revealing late gadolinium enhancement of the endomyocardium at the left ventricular apex.

**Figure 2:**
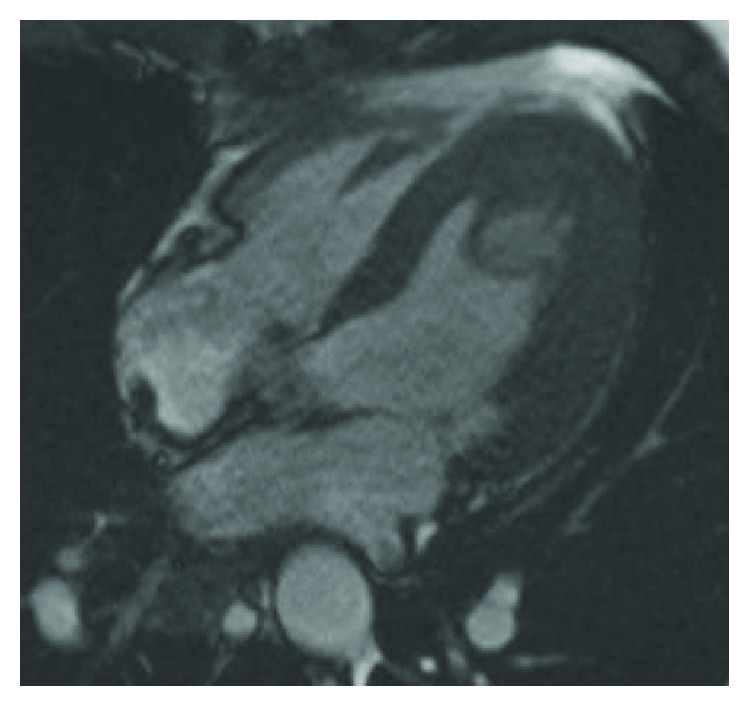
Cardiac magnetic resonance imaging 4-chamber view (still frame from a steady-state free precession (SSFP) cine sequence) with the left ventricular apical mass taken shortly after presentation.

His course was further complicated by development of left olecranon bursitis, treated by open drainage. The bursitis was culture negative but presumed septic. He also had several episodes of atypical chest pain. Electrocardiogram at the time of pain revealed sinus tachycardia, with lateral T wave inversion, and high-sensitivity troponin was elevated at 139 ng/L (NR < 26 ng/L). Transthoracic echocardiogram revealed normal left ventricular systolic function, with an echo-dense mass in the apex, no valvular abnormality, and mild left atrial dilatation. The patient proceeded to cardiac magnetic resonance (CMR) imaging which showed increased wall thickness in the mid to apical wall segments and high signal intensity in the mid to apical endocardium on short tau inversion recovery (STIR) imaging. There was late gadolinium enhancement in the same area, consistent with oedema in the apical endomyocardium (Figure 1). There was also a nonenhancing mass in the left ventricular apex consistent with thrombus and small bilateral pleural effusions (Figure 2).

Anticoagulation with therapeutic dose enoxaparin was commenced, which was subsequently changed to apixaban 5 mg twice daily prior to discharge.

**Figure 3:**
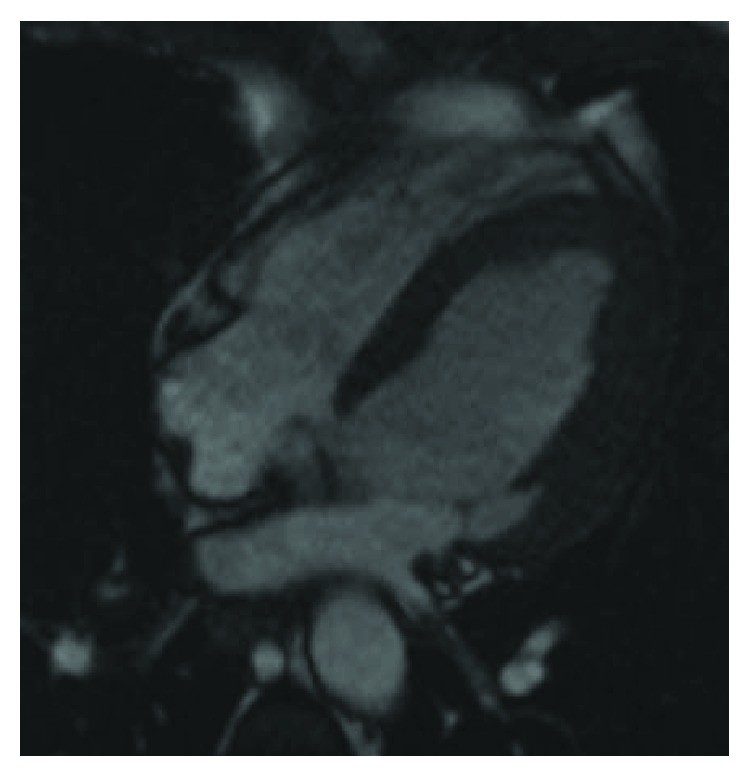
Cardiac magnetic resonance imaging 4-chamber view (still frame from a steady-state free precession (SSFP) cine sequence) taken 3 weeks after the previous study and on treatment (apixaban and antibiotics) demonstrating resolution of the apical mass.

A repeat echocardiogram was performed 10 days after the initial echocardiogram, which did not reveal any interval change. The patient was discharged with IV flucloxacillin to continue via a peripherally inserted central catheter (PICC) line and ongoing anticoagulation with apixaban. Repeat CMR was performed 3 weeks following discharge and revealed normal left ventricular cavity size, with mildly increased wall thickness in the mid to apical wall segments. Tissue characterisation was consistent with apical wall oedema, which had reduced significantly in size since the previous CMR, and complete resolution of the left ventricular apical mural thrombus and pleural effusions (Figure 3). He completed a 6-week course of IV flucloxacillin, followed by a 6-week course of oral dicloxacillin, and remained clinically well throughout this time.

## 3. Discussion

The majority of published cases of bacterial myocarditis are autopsy studies and predate the use of antibiotics [1]. Flaxman in 1943 described 17 cases of staphylococcal myocardial abscesses without endocarditis [7]. Sanders in 1963 reported nine similar cases [8]. A further seven cases of staphylococcal myocarditis have been reported, six of which died with diagnosis confirmed at autopsy [9–14]. One case had aortic valve insufficiency requiring valve replacement secondary to a massive intramyocardial abscess [15]. All cases developed septic shock as a result of staphylococcal bacteraemia, and one case died as a result of ventricular rupture [10]. Risk factors for infection were identified in four cases including end-stage renal disease on haemodialysis [12], steroid-dependent Crohn's disease, and initiation of infliximab [15] and two cases with AIDS [13].

This case demonstrates two aspects of myocarditis that are unusual: firstly, isolated *staphylococcus aureus* myocarditis with no evidence of valvular involvement and secondly, left ventricular apical thrombus formation in a patient with normal left ventricle systolic function.

Isolated *staphylococcus aureus* myocarditis remains a rare condition. There have been case reports of both methicillin-sensitive and methicillin-resistant infections. As distinct from endocarditis and device infections, these cases of myocarditis are almost exclusively described in individuals who are immunocompromised.

Several conditions can present with thrombus or thrombus-like formation in the left ventricular apex, including dilated cardiomyopathy, Loeffler's endocarditis, myxoma, Chagas disease, and aneurysms. Factors that influence thrombus formation include blood stagnation, endothelial injury, and hypercoagulable states [16, 17].

In this case, the predominant force of Virchow's triad is likely endothelial injury and inflammation secondary to myocarditis. Echocardiography demonstrated the apical mass but was not able to define the thrombus or associated inflammation in the myocardium, whereas CMR was useful for tissue characterisation but not able to identify the aetiology.

Anticoagulation in cases of left ventricular thrombus, especially in the context of normal systolic function, has limited evidence but is used to reduce the risk of embolisation. Warfarin has historically been used due to familiarity and lack of evidence with direct acting oral anticoagulants (DOACs). Several cases of successful treatment with DOACs have been described [18], but to our knowledge, this is the first case of thrombus treatment with apixaban in the context of normal systolic function.

## Figures and Tables

**Table 1:** Blood work on presentation to hospital and peak values.

	Presentation	Peak	Laboratory reference
White blood cell count	12.5	15.4	10^9^/L 4–11
Neutrophils	8.2	12.7	10^9^/L 2–8
Haemoglobin	162	162	g/L 130–180
Platelets	87	466	10^9^/L 150–400
Bicarbonate	17	25	mmol/L 22–32
Urea	16.3	16.3	mmol/L 3.5–8
Creatinine	290	290	*μ*mol/L 60–110
Bilirubin	69	69	*μ*mol/L < 20
GGT	70	70	U/L 5–50
ALP	76	105	U/L 30–100
ALT	69	69	U/L < 50
AST	74	92	U/L < 45
C-reactive protein	339	348	mg/L < 5
HS troponin		139	Ng/L < 26
